# Interpret Gaussian Process Models by Using Integrated Gradients

**DOI:** 10.1002/minf.202400051

**Published:** 2024-11-26

**Authors:** Fan Zhang, Naoaki Ono, Shigehiko Kanaya

**Affiliations:** ^1^ Division of Information Science Graduate School of Science and Technology Nara Institute of Science and Technology 8916-5 Takayama Ikoma, Nara 630-0192 Japan; ^2^ Data Science Center Graduate School of Science and Technology Nara Institute of Science and Technology 8916-5 Takayama Ikoma, Nara 630-0192 Japan

**Keywords:** explainable AI, gaussian process, integrated gradients

## Abstract

Gaussian process regression (GPR) is a nonparametric probabilistic model capable of computing not only the predicted mean but also the predicted standard deviation, which represents the confidence level of predictions. It offers great flexibility as it can be non‐linearized by designing the kernel function, made robust against outliers by altering the likelihood function, and extended to classification models. Recently, models combining deep learning with GPR, such as Deep Kernel Learning GPR, have been proposed and reported to achieve higher accuracy than GPR. However, due to its nonparametric nature, GPR is challenging to interpret. While Explainable AI (XAI) methods like LIME or kernel SHAP can interpret the predicted mean, interpreting the predicted standard deviation remains difficult. In this study, we propose a novel method to interpret the prediction of GPR by evaluating the importance of explanatory variables. We have incorporated the GPR model with the Integrated Gradients (IG) method to assess the contribution of each feature to the prediction. By evaluating the standard deviation of the posterior distribution, we show that the IG approach provides a detailed decomposition of the predictive uncertainty, attributing it to the uncertainty in individual feature contributions. This methodology not only highlights the variables that are most influential in the prediction but also provides insights into the reliability of the model by quantifying the uncertainty associated with each feature. Through this, we can obtain a deeper understanding of the model′s behavior and foster trust in its predictions, especially in domains where interpretability is as crucial as accuracy.

## Graphical abstract

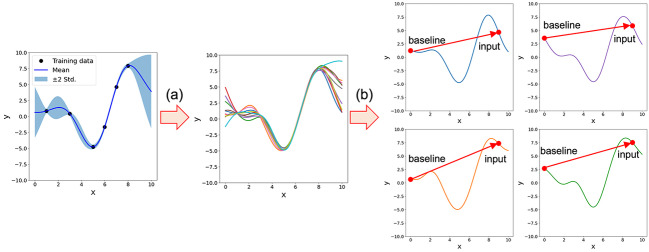

## 1 Introduction

Gaussian process regression (GPR) is a nonparametric probabilistic model that not only predicts values but also calculates the predicted standard deviation, signifying confidence in its predictions. Its flexibility is marked by the capability to non‐linearize through kernel function design, enhance outlier robustness by modifying the likelihood function, and expand to classification models [1]. Thus, GPR have been extensively applied in material science, drug discovery research and formulation development [2, 3, 4]. In recent times, advanced models such as Deep Kernel Learning Gaussian processes Regression (DKLGPR) which combine neural networks (NN) with GPR, have been proposed, reporting enhanced modeling precision over conventional GPR [5]. However, the nonparametric nature of GPR complicates its interpretability, a growing demand in the field for Explainable AI, which is called Explainable AI (XAI), from various perspectives, encompassing both global and local interpretability. In terms of global interpretability, GPR can be interpreted by comparing kernel function scale parameters or Automatic Relevance Determination (ARD) kernel length parameters. It has been noted that ARD kernels may undervalue explanatory variables linearly related to the target variable [6], leading to the proposal of a feature selection method through sensitivity analysis of the posterior distribution [7]. Conversely, many model‐agnostic methods for local interpretation have been proposed recently. Techniques employing interpretable surrogate models like LIME [8] or kernel SHAP [9] exist but come with the drawbacks of randomness in outcomes and high computational costs for high‐dimensional data. Sensitivity analysis also be used; however, it does not satisfy the axioms of Sensitivity and Implementation Invariance, prompting the introduction of Integrated Gradients (IG), widely used in interpreting deep learning models [10]. Yet, these methods, when applied as is, struggle to interpret the predicted standard deviation of GPR. Probabilistic models similar to GPR, such as GPX, have been developed for interpretability [11], but their applicability to combining with deep learning and to classification problems has not been sufficiently validated. Therefore, this study reports the development of an IG‐based method capable of interpreting not only GPR but also models like DKLGPR and GPC, addressing the interpretative challenges of both predicted mean and standard deviation.

## 2 Background

### 2.1 Gaussian Process Regression (GPR)

GPR sets the prior distribution of the latent function f as specified in Eq. (1), and the observed values y are generated according to Eq. (2). Here, X is the matrix of explanatory variables, $m_{n}$ is a vector consisting of mn copies of the constant m, and σ is a constant. Consequently, the latent function and the predicted distribution for a new input 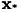 can be calculated as delineated in Eqs. (3) and (4). Here, K represents the kernel matrix for the training data, 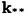 for the test data, and 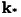 between the training and test data. The hyperparameters of the kernel function, m and $\sigma^{2}$ can be optimized to maximize the marginal likelihood.

(1) $$p\left(f|X\right)=\;𝒩\left(m_{n},\;K\right)$$

(2) $$p\left(y|f\right)=\;𝒩\left(f,\;\sigma^{2}I\right)$$

(3)

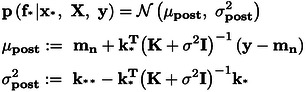



(4)

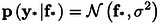




### 2.2 Scalable Variational Gaussian Process Regression (SVGPR)

One of the drawbacks of GPR is that when the number of training data points is N, the computational cost is $𝒪\left(N^{3}\right)$ which makes it challenging to train with large‐scale data. Recently, SVGPR, which uses M inducing points, has been proposed [12]. This method can reduce the computational cost to $𝒪\left({NM}^{2}\right)$ . The setup for SVGPR is as follows: let $f_{n}$ be the latent function at n‐th input $x_{n}$ and u be the latent function at inducing inputs z , then u , $f_{n}$ , and n‐th outcome $y_{n}$ are generated according to Eqs. (5) to (7). Here, $K_{zz}$ represents the kernel matrix for the inducing points, $k_{xx}$ for the train data, and 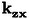 between the inducing points and training data. Consequently, the latent function and the predicted distribution for a new input 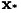 can be computed using Eqs. (8) and (9). The inducing inputs z, as well as the parameters and hyperparameters of GPR, can be optimized using variational inference [13, 14].

(5) $$p\left(u\right)=\;𝒩\left(m_{n},\;K_{zz}\right)$$

(6) $$p\left(f_{n}|u\right)=\;𝒩\left(\mu_{zpost},k_{xx}-k_{zx}^{T}{K_{zz}}^{-1}k_{zx}\;\right)\mu_{zpost}:=\;m_{n}+k_{zx}{K_{zz}}^{-1}\left(u-m_{n}\right)$$

(7) $$\left(y_{n}|f_{n}\right)=\;𝒩\left(f_{n},\;\sigma^{2}\right)$$

(8)

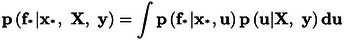



(9)

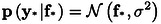




### 2.3 Deep Kernel Learning Gaussian Process Regression (DKLGPR)

A model that enhances the expressive capability of GPR is the DKLGPR, which incorporates NN. This approach utilizes NN to transform the input x into latent variables ϕ , as described by Eqs. (10) and (11). The variable $h^{\left(i\right)}$ represents the vector at the i‐th layer, while $W_{i}$ and $b_{i}$ denote the weight matrix and bias vector at the i th layer, respectively. The function g represents an activation function. The variable ϕ follows a GPR model, as demonstrated by Eqs. (12) and (13). In this study, LeakyReLU, which is described by Eq. (14), with α=0.1 was employed. The predicted distribution can be computed in accordance with Eqs. (15) and (16). Φ is a matrix consisting of ϕ , and 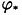 is a vector representing the test data transformed using the NN. Here, $K_{\phi }$ represents the kernel matrix for the transformed training data, 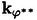 for the transformed test data, and 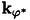 between the transformed training data and transformed test data. The parameters of the NN and GPR, as well as the hyperparameters of the GPR, can be optimized to maximize the marginal likelihood.

(10) $$h^{\left(i+1\right)}=\;g\left(W_{i}h^{\left(i\right)}+b_{i}\right)$$

(11) $$=\;g\left(W_{l}h^{\left(l\right)}+b_{l}\right)$$

(12) $$p\left(f|\Phi \right)=\;𝒩\left(m_{n},\;K_{\phi }\right)$$

(13) $$p\left(y|f\right)=\;𝒩\left(f,\;\sigma^{2}I\right)$$

(14) $$LeakyReLU\left(x;\;\alpha \right)=\left\{\begin{array}{c} \;x,\;\;\;x\geq 0\;\\ \;\alpha x,\;x\leq 0\; \end{array}\right.$$

(15)

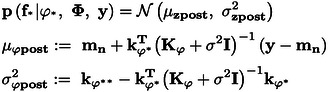



(16)

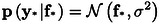




### 2.4 Gaussian Process Classification (GPC)

GPC posits that the latent function f of input X follows the form of Eq. (17), and the $y_{n}$ adheres to a Bernoulli distribution parameterized by $\sigma \left(f_{n}\right)$ , as described by Eqs. (18) and (19). Since $p\left(f|X,\;y\right)$ cannot be computed analytically, it is necessary to approximate it with $q\left(f\right)$ , as indicated in Eq. (20). **μ** and **Σ** represent the mean vector and covariance matrix, respectively. Utilizing $q\left(f\right)$ , the latent function and the predicted distribution for a new input 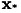 can be computed as shown in Eqs. (21) and (22). The hyperparameters of the kernel function and the approximate distribution $q\left(f\right)$ can be optimized to maximize the Evidence Lower Bound (ELBO). Similar to GPR, GPC can also utilize inducing points to improve computational efficiency [15], a method we refer to as Scalable Variational Gaussian Process Classification (SVGPC).

(17) $$p\left(f\right)=\;𝒩\left(m_{n},\;K\right)$$

(18) $$p\left(y_{n}=1|f_{n}\right)=\;\sigma \left(f_{n}\right)$$

(19) $$\sigma \left(x\right)=\;\frac{1}{1+e^{-x}}$$

(20) $$p\left(f|X,\;y\right)\approx q\left(f\right)=\;𝒩\left(\mu ,\;\Sigma \right)$$

(21)

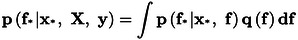



(22)

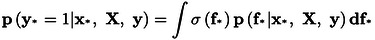




### 2.5 Integrated Gradients (IG)

As a method that satisfies the axioms of Sensitivity and Implementation Invariance, which are essential for Explainable AI, IG has been proposed [10]. IG can be understood as averaging the gradient of the output f with respect to feature i along the path from a baseline 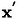 to x , as shown in Eq. (23), and weighting it by the difference from the baseline. $x_{i}$ represents the feature i of x .
(23)

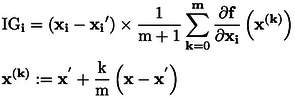




## 3 Proposed Method

**Figure 1:**
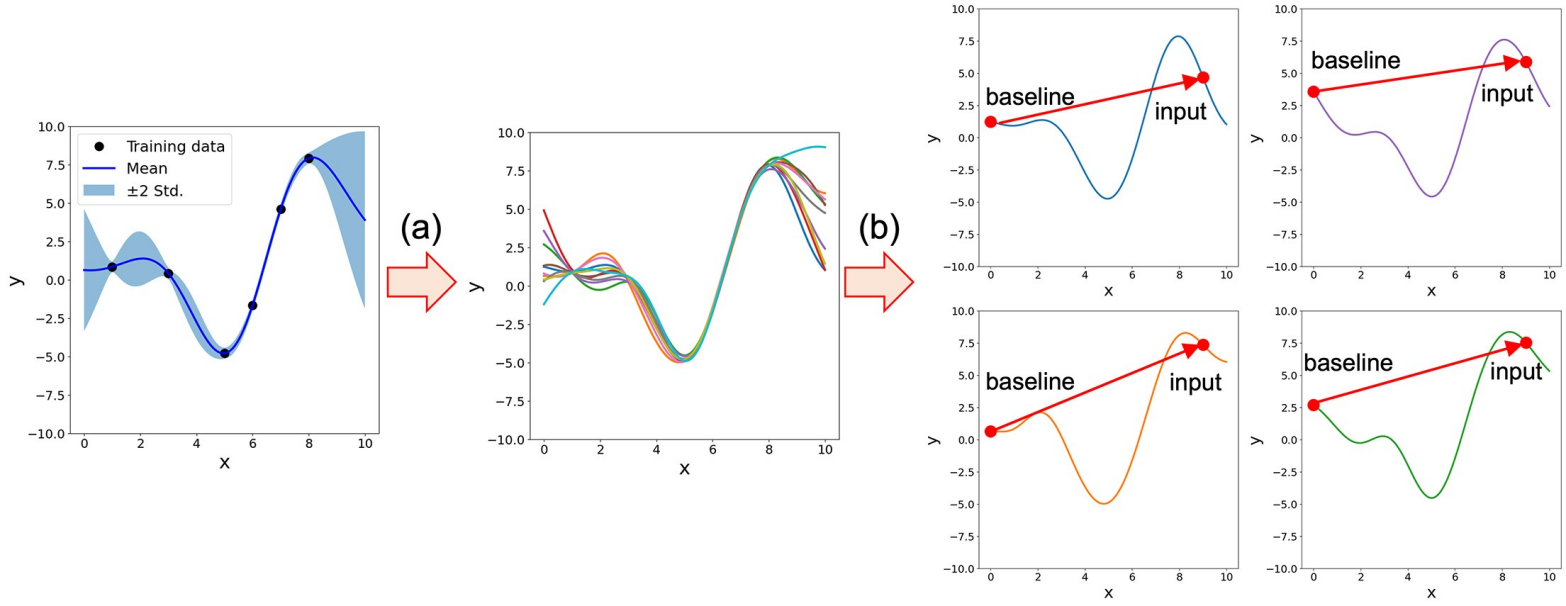
The scheme of proposed method. (a) Sampling of latent functions from the posterior distribution, (b) Interpretation of each function by using IG.

Gaussian Processes models can be considered probabilistic models that generate functions. Therefore, we hypothesized that by sampling multiple latent functions from the Gaussian Process models and interpreting each one, we can infer the meaning behind the Gaussian Process′s predicted mean and standard deviation. For the interpretation of the latent functions, we used IG, which satisfies the axioms of interpretability methods. The latent functions from the baseline to the desired input can be sampled from 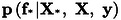 , as described by Eq. (24). 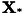 is a matrix that collects the vectors from the baseline to the desired input. The expected value of the IG, as described by Eq. (25), with respect to the feature i can be viewed as the contribution of the feature i to the mean of the predicted distribution. Similarly, the standard deviation of IG, as described by Eq. (26), represents the contribution to the standard deviation of the predicted distribution. The scheme of the proposed method is shown in Figure 1.
(24)

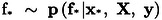



(25)

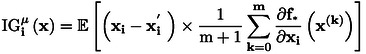



(26)

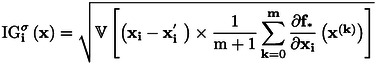




However, since 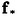 is a random variable, it cannot be differentiated. Using an infinitesimal amount ϵ and the unit vector $e_{i}$ where the feature i is 1, and by taking 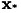 as indicated in Eq. (27), the sampled latent functions are as shown in Eq. (28), which allows for numerical differentiation. 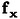 represents the value of the function f at x . Contributions to the predicted mean and standard deviation for each feature can then be calculated from Eqs. (29) and 30.
(27)

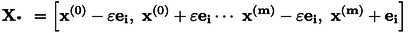



(28)

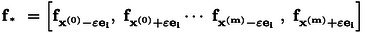



(29)

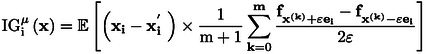



(30)

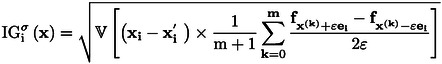




This method is highly versatile as it can be applied to both GPR and GPC without restriction on the kernel function used or on the architecture of the neural network in DKLGPR, provided that 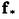 can be sampled.

## 4 Experiments

### 4.1 Settings

In this study, we conducted experiments using the Radial Basis Function (RBF) kernel as defined by the Eq. (31). $x^{\left(i\right)}$ and $x^{\left(j\right)}$ indicate data points and $\theta_{0}$ and $\theta_{1}$ are hyperparameters.

(31) $$k\left(x^{\left(i\right)},x^{\left(j\right)}\right)=\theta_{0}exp\left(-\frac{{∥x^{\left(i\right)}-x^{\left(j\right)}∥}^{2}}{\theta_{1}}\right)$$

For the inducing points method, we set the number of inducing points to 200. The IG were calculated with m=100 , ϵ=0.001 and the latent functions were sampled 1000 times. All explanatory variables were standardized, in the case of regression, the target variable was also standardized before being used for training. All parameters and hyperparameters of the GPR models were trained using the Adam [16] optimizer with a learning rate of 0.1 for 1000 iterations. For the DKLGPR models, the parameters and hyperparameters were trained using the Adam optimizer with a learning rate of 0.01 for 1000 iterations. The SVGPR models were trained for 1000 iterations using Natural Gradient Descent (NGD) [17] for the parameters with a learning rate of 0.1, and the Adam optimizer for the hyperparameters with a learning rate of 0.01. The SVGPC model was trained for 1000 iterations using the Adam optimizer with a learning rate of 0.01 for both parameters and hyperparameters. The models trained using variational inference employed parameters and hyperparameters that minimized the loss function. During Bayesian optimization, the Gaussian process was trained with an additional configuration that reduced the learning rate whenever the monitored metric stopped improving. The reduction factor was set to 0.5, with a minimum learning rate of 0.0001, and the learning rate was decreased after three epochs without improvement.

### 4.2 Data

**Figure 2:**
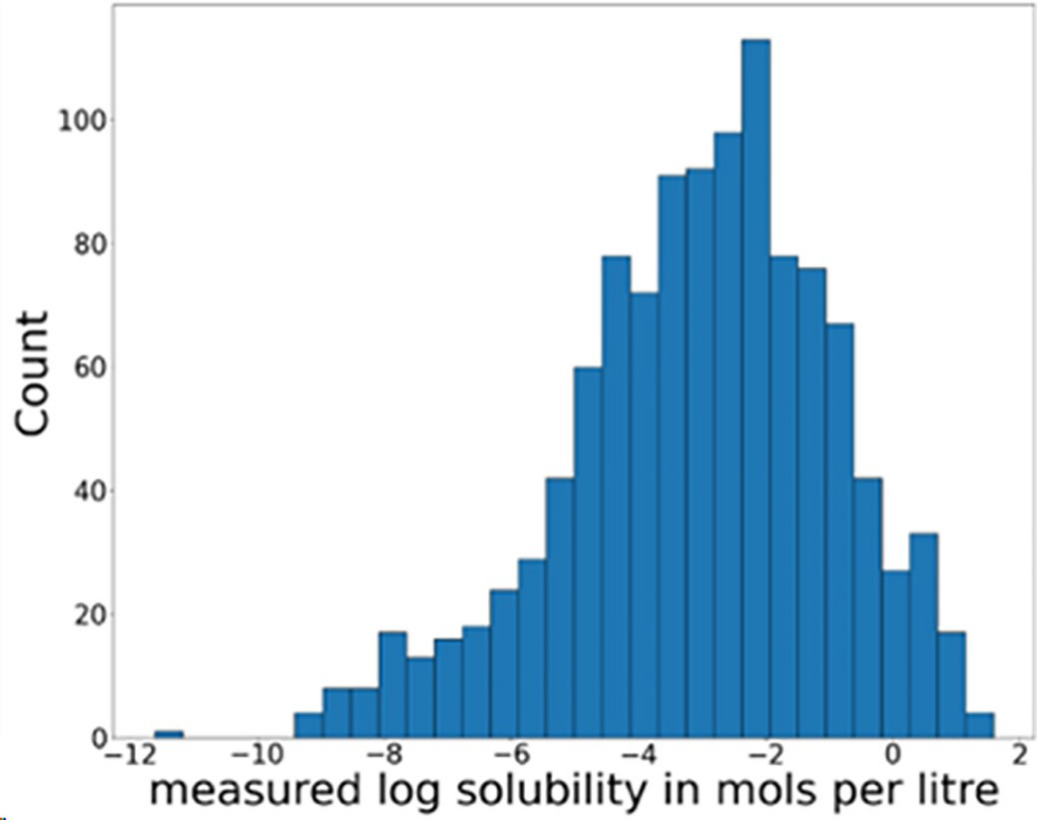
Histogram of measured log solubility in mols per liter.

We utilized the dataset that contains solubility data for about 1100 organic compounds in water. [18] The distribution of this data is illustrated in Figure 2. It is known that organic compounds with highly polar functional groups, such as hydroxyl groups, have increased solubility in water, while those with low polarity functional groups, like benzene rings, have decreased solubility. This characteristic makes it easier to assess the correctness of interpretability, which is why we chose this dataset. We designated a random set of 200 data points from the dataset as the evaluation data and used the remaining data for training.

### 4.3 Implementation

GPytorch [19] was used for model construction. RDKit [20] was utilized for molecular feature calculation and visualization.

### 4.4 Interpret the GPR

**Figure 3:**
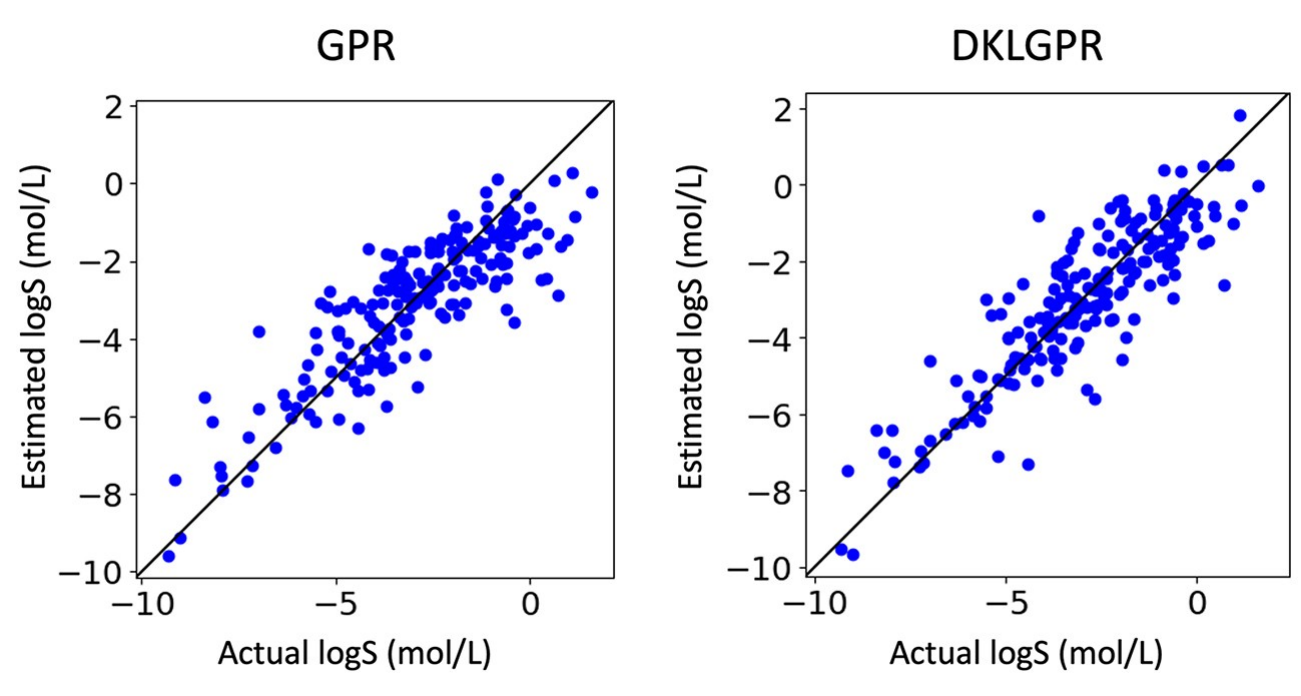
Actual logS (mol/L) v.s. Estimated logS (mol/L).

**Figure 4:**
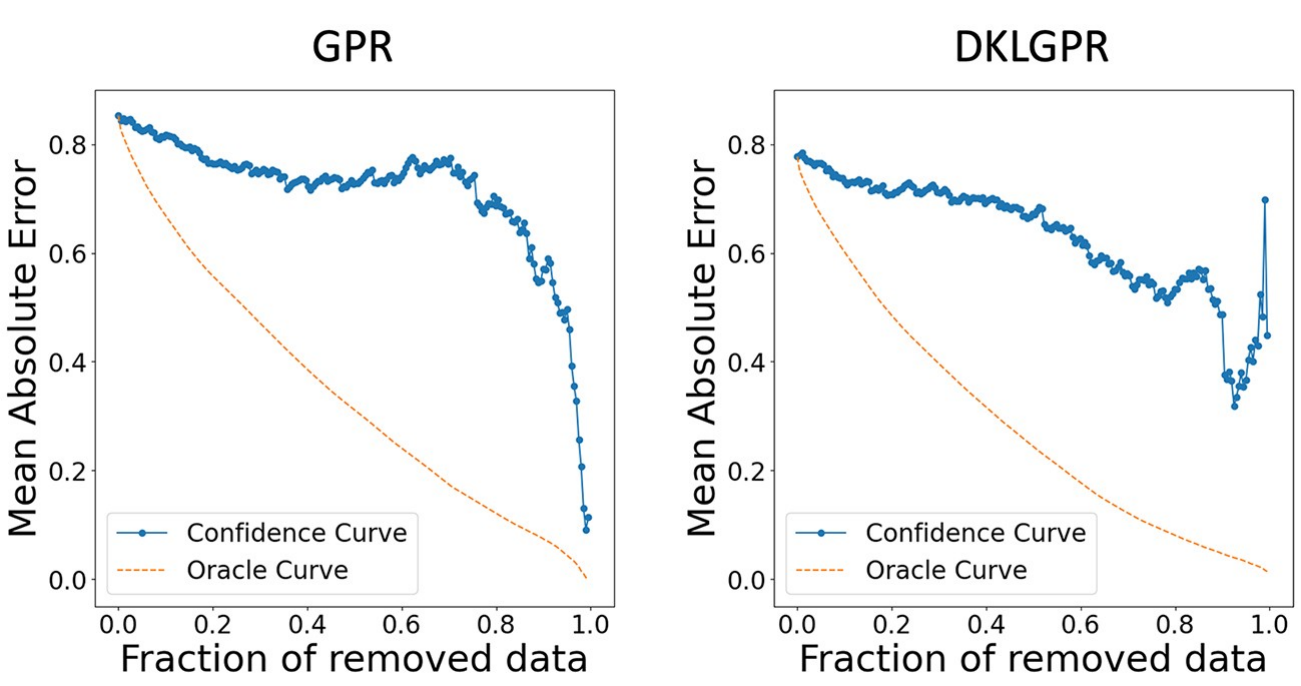
Confidence oracle curve.

For the representation of compounds, we employed count‐based Extended‐Connectivity Fingerprints 4 (CECFP4) which counts the ECFP [21] substructures with a radius of 2 bonds around each atom in a molecule. Features where of 0.95 or higher of the values were the same across the dataset were removed, and from pairs with a correlation coefficient more than 95%, one was eliminated. We used a set of 200 data points as training data to construct both GPR and DKLGPR models, and evaluated them using the evaluation data. The NN for DKLGPR was configured with intermediate layer nodes set to (128, 64). The results are presented below (Table 1, Figures 3 and 4). We calculated the coefficient of determination (R^2^), root mean squared error (RMSE), mean absolute error (MAE), and the area under the oracle confidence error (AUCO) to evaluate regression model performance. AUCO is a metric to quantify the accuracy of predictive uncertainty [22, 23]. AUCO is defined by the Eq. (31), and a smaller value indicates better performance.

(32) $$AUCO\;=\frac{1}{N-1}\sum_{n=1}^{N-1}\left({MAE}_{n}^{conf}-{MAE}_{n}^{orac}\right)$$

**Table 1:** Prediction accuracy of GPR and DKLGPR trained with 200 data.

Model	GPR	DKLGPR
R^2^	0.737	0.769
RMSE	1.11	1.05
MAE	0.853	0.777
AUCO	0.368	0.346

**Figure 5:**
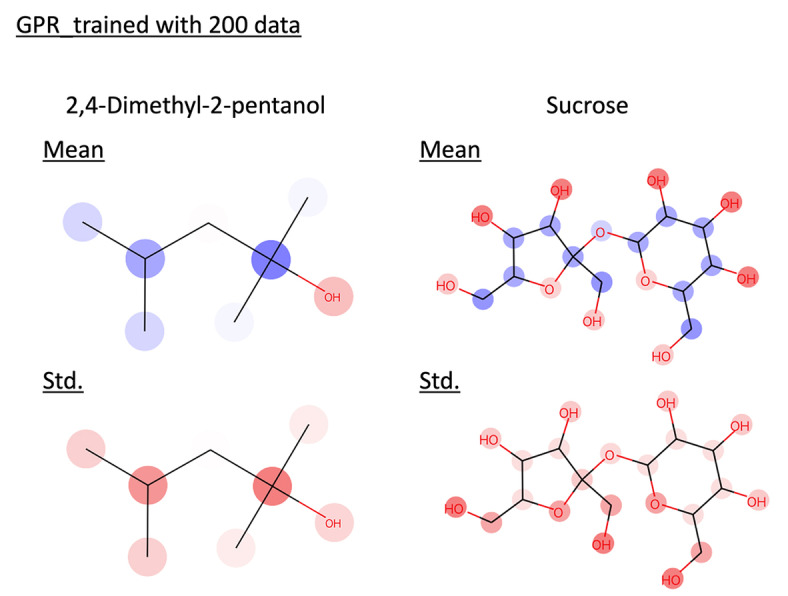
Interpret predicted mean and standard deviation of GPR trained with 200 data by the proposed method. Red represents positive values, blue represents negative values, and the color intensity indicates the value magnitude.

**Figure 6:**
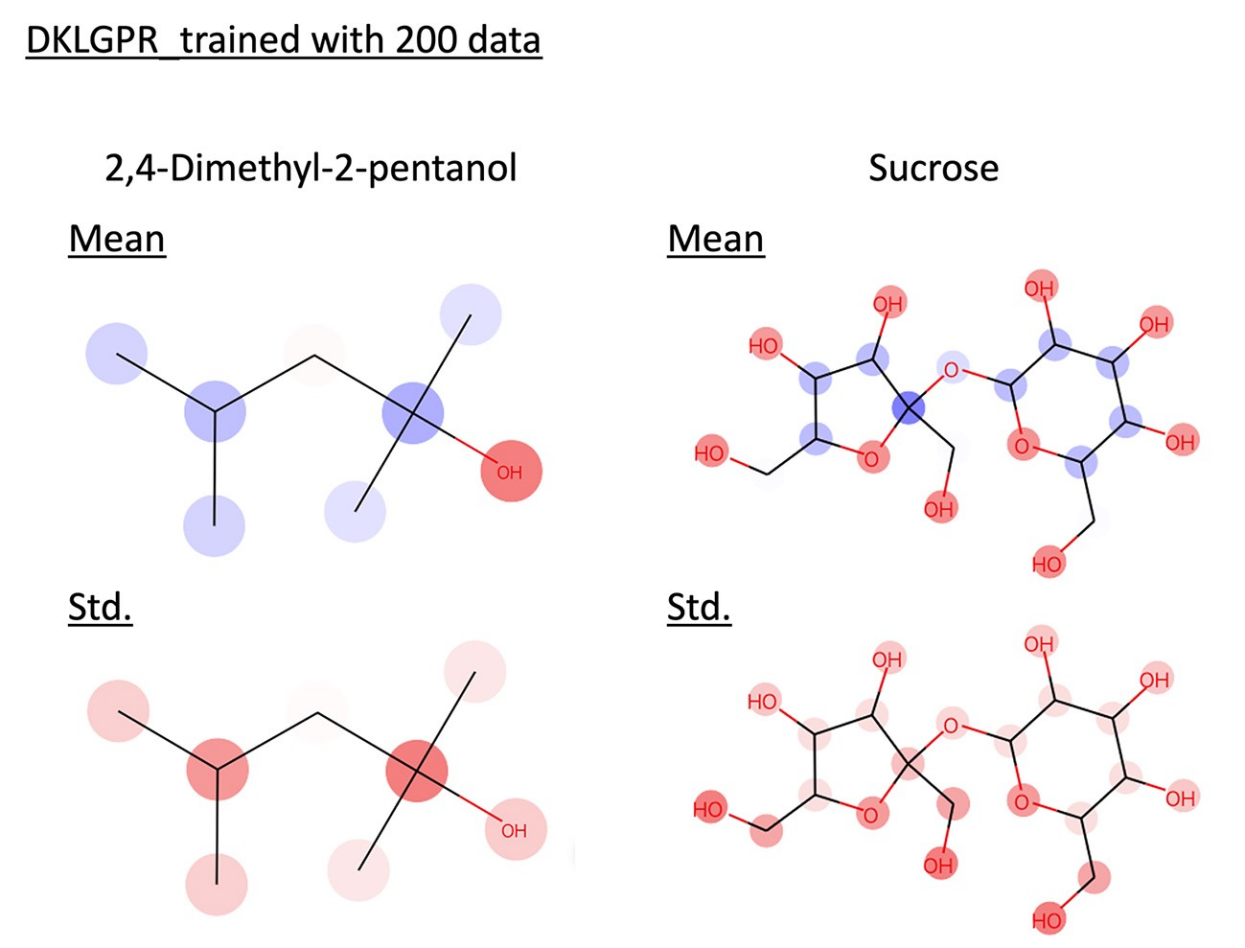
Interpret predicted mean and standard deviation of DKLGPR trained with 200 data by the proposed method. Red represents positive values, blue represents negative values, and the color intensity indicates the value magnitude.

${MAE}_{n}^{orac}$ is calculated by computing the absolute error between the observed and predicted values, sorting them in ascending order, removing the lowest n data points, and then averaging the remaining values. ${MAE}_{n}^{conf}$ is calculated by sorting the absolute errors between the observed and predicted values based on the predicted uncertainty (in this case, the standard deviation of 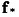 prediction), removing the lowest n data points, and then averaging the remaining values. Plotting ${MAE}_{n}^{orac}$ against n yields the oracle curve, while plotting ${MAE}_{n}^{conf}$ against n yields the confidence curve. Both models achieved $R^{2}$ value higher than 0.7, confirming their ability to make accurate predictions. The superior predictive performance of DKLGPR is attributed to the increased expressive power of the model resulting from the combination of NN with GPR. GPR assumes that data points with similar explanatory variables will have similar target variables. However, it does not assume that data points with dissimilar explanatory variables will have dissimilar target variables. The predicted standard deviation of GPR is calculated based on the similarity to the training data. Therefore, it is expected that removing data points with high predictive standard deviation will not significantly reduce the MAE. In fact, by plotting the confidence curve, we confirmed this characteristic, as it showed a sharp decline in the latter part. Using the proposed method, we interpreted the prediction results for 2,4‐Dimethyl‐2‐pentanol and sucrose in these models (Figure 5 and 6). In the interpretation of the predicted mean, red indicates an increase in solubility in water, while blue indicates a decrease, with the color intensity representing the value magnitude. In both GPR and DKLGPR models, alkyl groups are colored blue, and hydroxyl groups are colored red, aligning with scientific knowledge in the interpretative results. Furthermore, the interpretation of the predicted standard deviation indicates that a darker color represents higher uncertainty. Quaternary carbons and hydroxymethyl groups can be interpreted as having high uncertainty. To improve the accuracy of the model′s interpretation, it is possible that further experimentation with compounds containing quaternary carbons or hydroxymethyl would be beneficial.

**Figure 7:**
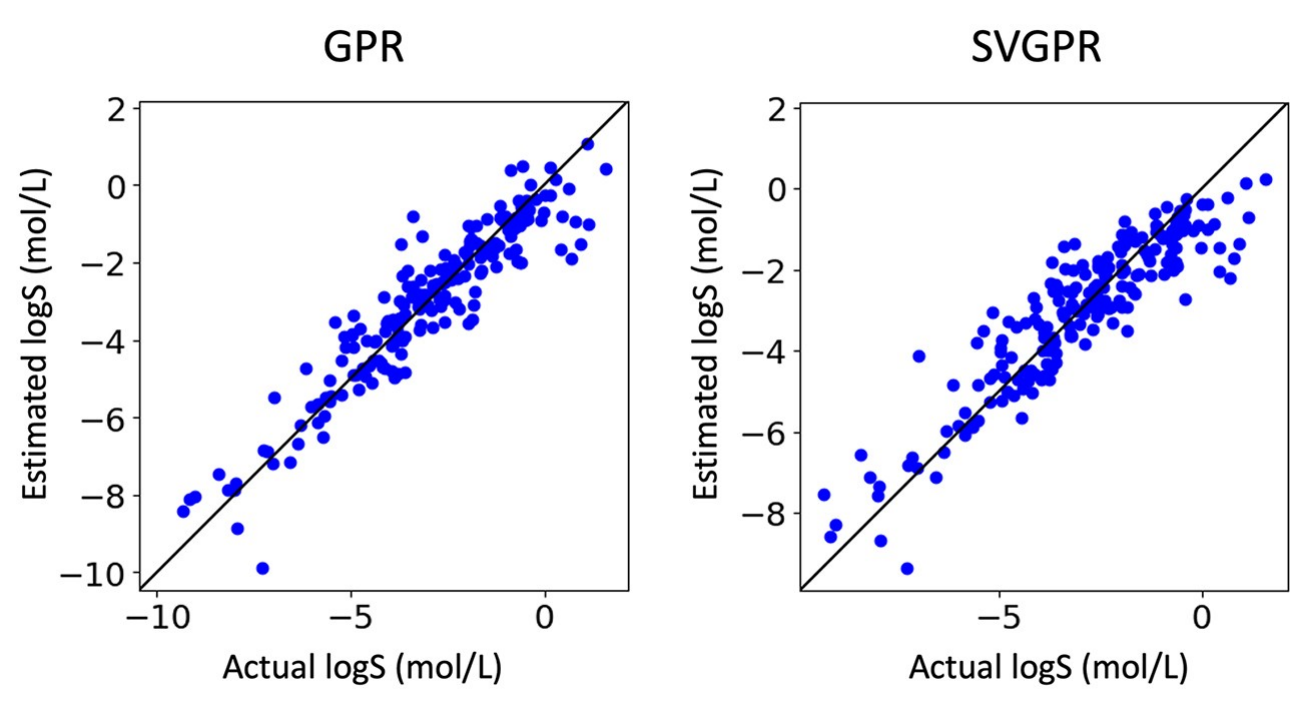
Actual logS (mol/L) v.s. Estimated logS (mol/L).

**Figure 8:**
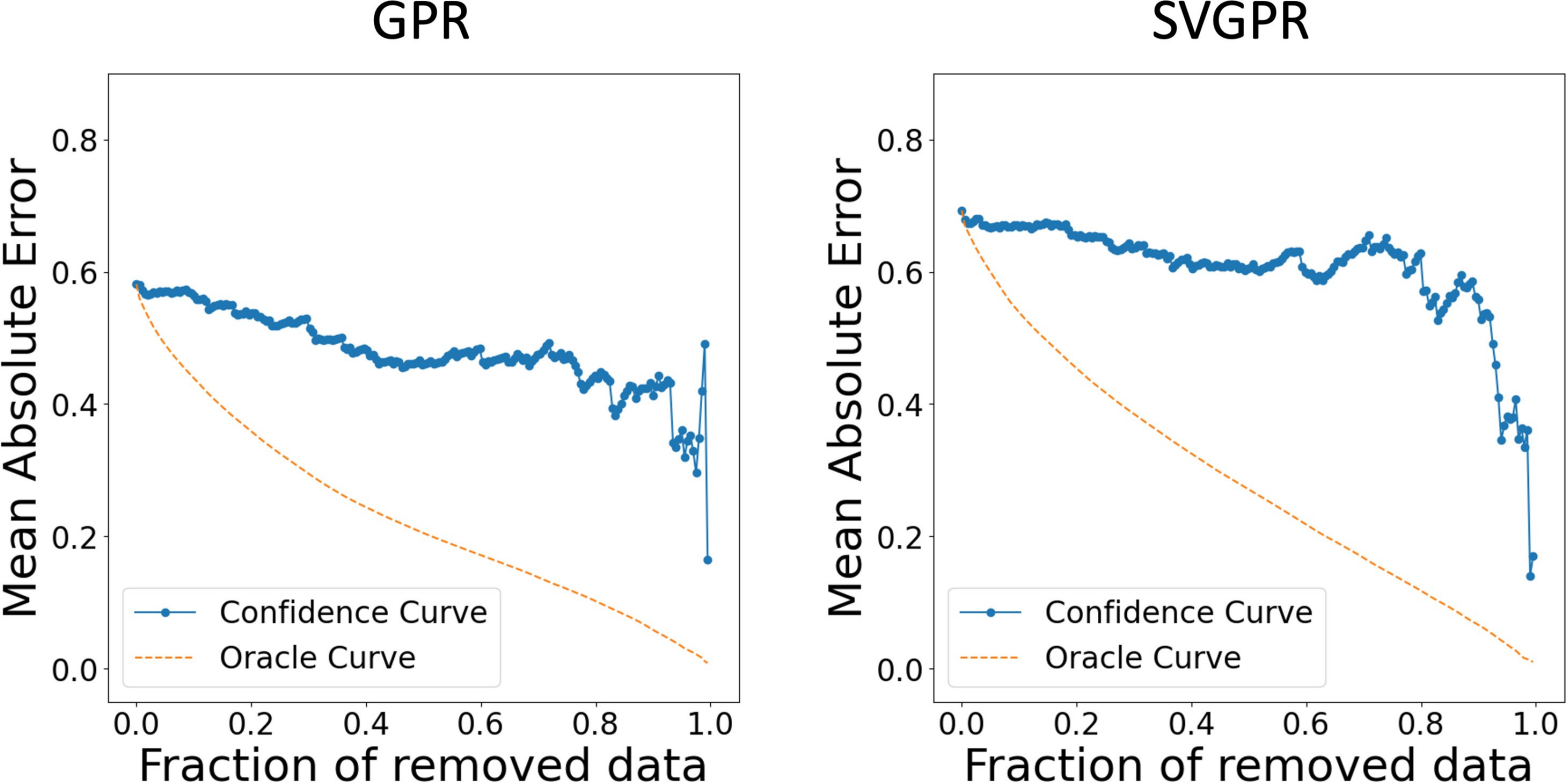
Confidence oracle curve.

**Figure 9:**
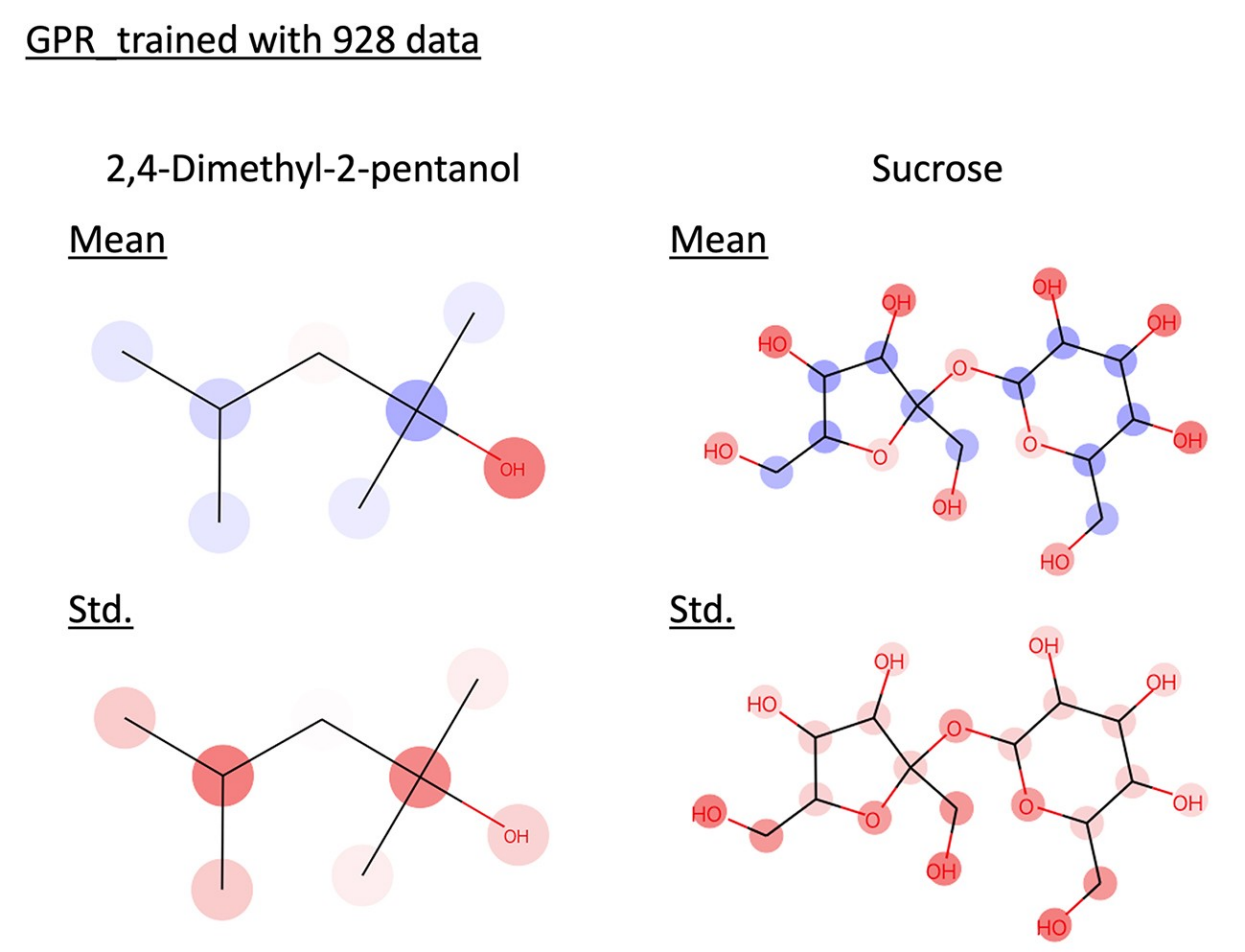
Interpret predicted mean and standard deviation of GPR trained with 928 data by the proposed method. Red represents positive values, blue represents negative values, and the color intensity indicates the value magnitude.

**Figure 10:**
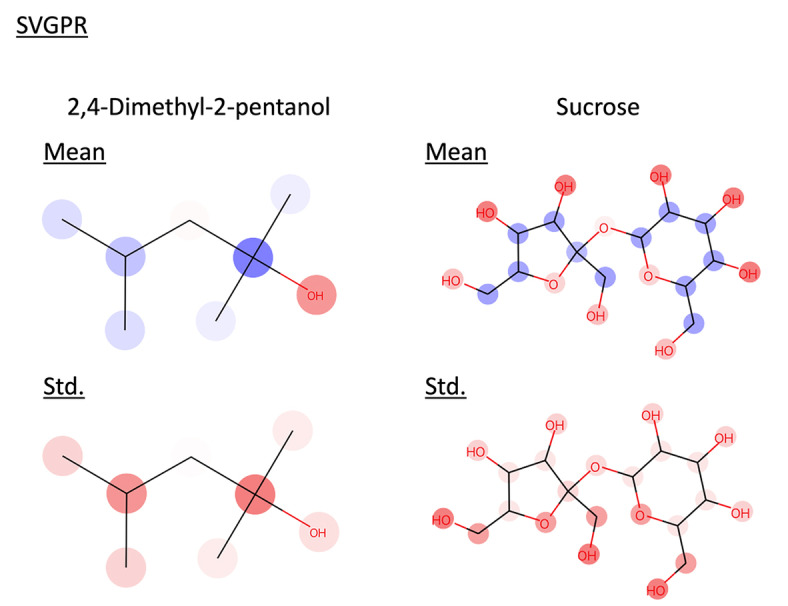
Interpret predicted mean and standard deviation of SVGPR trained with 928 data by the proposed method. Red represents positive values, blue represents negative values, and the color intensity indicates the value magnitude.

GPR faces increased computational costs as the number of training data grows, which becomes a bottleneck. SVGPR is a method proposed to address this issue. In this context, we verified whether SVGPR could be interpreted. Both GPR and SVGPR were trained using 928 data points. The features used were the same as those used when training with 200 data points. The results are shown below (Table 2, Figure 7, and 8). Both models achieved an R^2^ value higher than 0.8, confirming their ability to make accurate predictions. Similarly, the confidence curves of the SVGPR model, like those of the GPR model, showed a significant drop in the latter part. We interpreted the prediction results of these models for 2,4‐Dimethyl‐2‐pentanol and sucrose (Figure 9 and 10). It was confirmed that SVGPR can be correctly interpreted similarly to GPR, and the proposed method can be correctly applied to models that perform approximate calculations using the inducing points method.

**Table 2:** Prediction accuracy of GPR and SVGPR trained with 928 data.

Model	GPR	SVGPR
R^2^	0.868	0.827
RMSE	0.791	0.903
MAE	0.582	0.693
AUCO	0.250	0.315

### 4.5 Interpret the GPC

**Figure 11:**
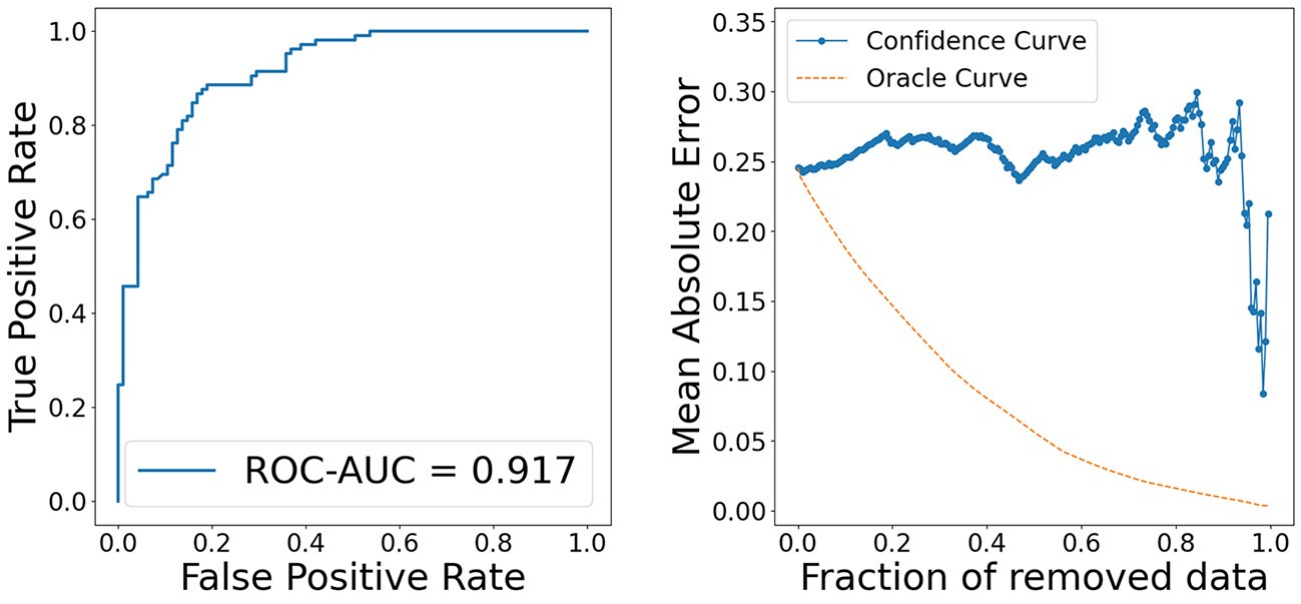
ROC‐AUC and Confidence Oracle curve.

**Figure 12:**
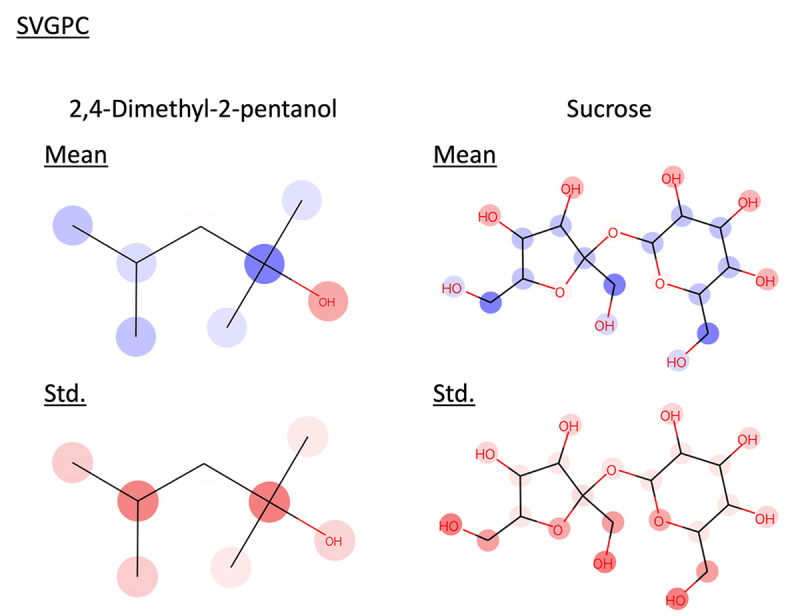
Interpret predicted mean and standard deviation of SVGPC trained with 928 data by the proposed method. Red represents positive values, blue represents negative values, and the color intensity indicates the value magnitude.

**Figure 13:**
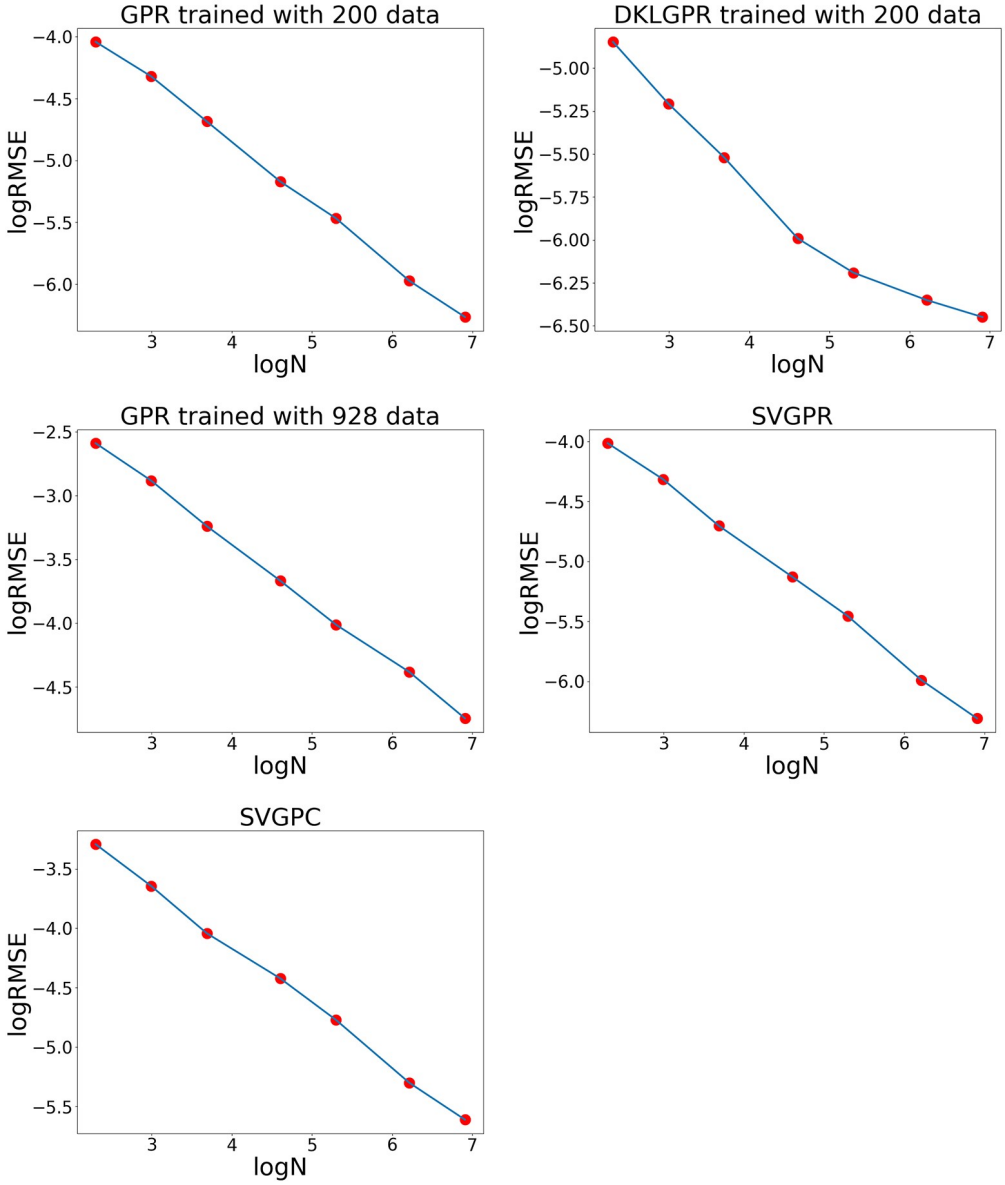
Convergence of the proposed method.

GPC is a model that modifies the likelihood function of GPR from Gaussian distribution to Bernoulli distribution. In this study, we defined instances where the measured log solubility in mols per litre was greater than −3 as 1, and those less than or equal to −3 as 0, and performed classification tasks using SVGPC. The model was trained using 928 data points, employing the same features as those used when training GPR with 200 data. The results are presented below (Table 3, Figure 11). We calculated Accuracy, Recall, Precision, F1‐score, Receiver Operating Characteristic Area Under the Curve (ROC‐AUC) and the Area Under the Confidence Oracle curve (AUCO) to evaluate classification model performance. For the AUCO in classification problems, MAE was calculated using the absolute error between the labels and the predicted probabilities 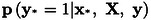 . The model attained F1‐scores exceeding 0.8, verifying their capability to generate accurate predictions. Similarly, the SVGPC model, like the GPR model, showed a significant drop in their confidence curves in the latter part. We interpreted the prediction results of these models for 2,4‐Dimethyl‐2‐pentanol and sucrose (Figure 12, 13). The interpretations for 2,4‐Dimethyl‐2‐pentanol and sucrose were almost correct. Although the interpretation of the predicted mean for structure′s hydroxymethyl groups in sucrose was incorrect, a look at the predicted standard deviation interpretation revealed it to be large. This indicates that the proposed method can also calculate which feature effects are uncertain within the model.

**Table 3:** Prediction accuracy of SVGPC trained with 928 data.

Model	SVGPC
Accuracy	0.845
Recall	0.886
Precision	0.830
F1‐score	0.857
ROC‐AUC	0.917
AUCO	0.176

### 4.6 Convergence of the Proposed Method

In the proposed method, we interpret the model by sampling latent variables. We examine the number of latent functions that should be sampled for adequate interpretation. Let 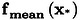 be the mean of 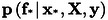 ; as the number of samples increases, the value given by Eq. (29) should converge to Eq. (33). We define the difference between ${IG}_{i}^{\mu }\left(x\right)$ and ${IG}_{i}\left(x\right)$ for each data point as per Eq. (34) and evaluate the convergence by calculating this difference for a subset from the evaluation data, changing the number of samples and taking the average. In Eq. (34), d represents the number of features, and n represents the number of samples used to calculate, which is 100 in this case.
(33)

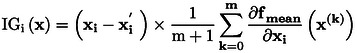

(34) $$RMSE=\frac{1}{n}\sum_{j=1}^{n}\sqrt{\frac{1}{d}\sum_{i=1}^{d}{\left({IG}_{i}\left(x\right){-IG}_{i}^{\mu }\left(x\right)\right)}^{2}}$$

We computed this for models trained on 200 data points for GPR, DKLGPR, and on 928 data points for SVGPR, and SVGPC (Figure 15). For all models, the $log\left(RMSE\right)$ decreased proportionally to $log\left(N\right)$ , which is consistent with the central limit theorem. This suggests that by sampling the latent functions f multiple times and plotting the $log\left(RMSE\right)$ , one can determine the number of samples required for interpretation with any desired level of precision.

### 4.7 Interpret Bayesian Optimization

As an application of GPR, Bayesian optimization can be employed. It is anticipated that the proposed method will allow us to interpret which variables Bayesian optimization focuses on and how it conducts the search. In this study, all molecules were vectorized using CECFP4, variables with more than 95% identical values were removed, and one of each pair of variables with a correlation coefficient of 0.95 or higher was eliminated. To ensure that none of the feature variances became zero, 30 data points were randomly selected from the 500 data points with the smallest target variable values and used as the initial data. Subsequently, we trained a GPR using these molecules and calculated the acquisition function for all remaining molecules, iteratively adding the molecule that maximized the acquisition function to the training data. The acquisition function used here is the Upper Confidence Bound (UCB) defined by Eq. 35.

(35) $$UCB\left(x\right)=\mu \left(x\right)+\alpha \times \sigma \left(x\right)$$

**Figure 14:**
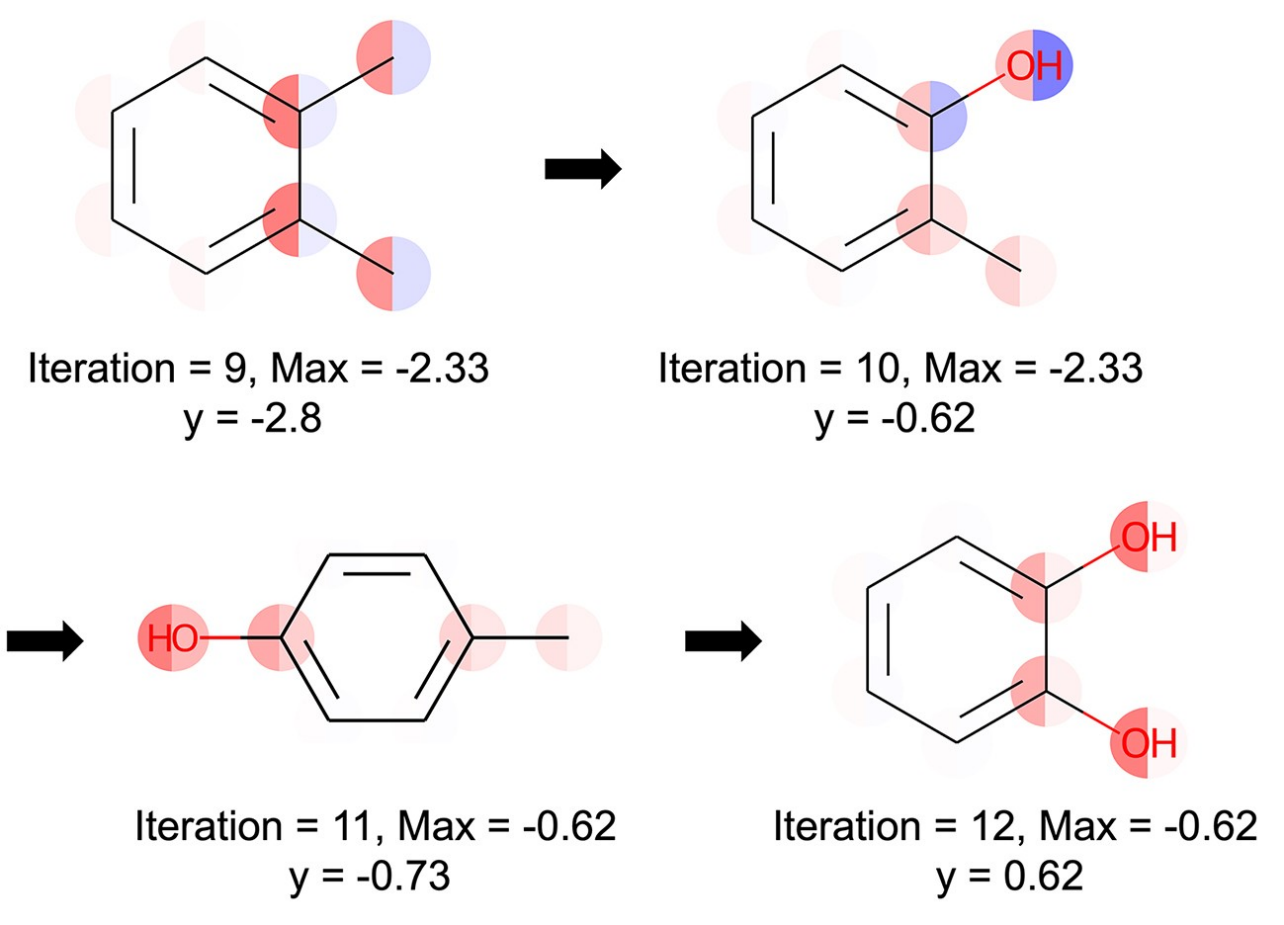
Interpret Bayesian optimization using the proposed method. Red represents positive values, blue represents negative values, and the color intensity indicates the value magnitude. Max represents the maximum value obtained from previous explorations, Iteration denotes the number of explorations conducted, and y is the property value of the compound evaluated. During the initial analysis, the oxygen atom′s halves were colored differently, obscuring its contribution. In the third iteration, both halves turned red, indicating a learned positive contribution.

Here, $\mu \left(x\right)$ and $\sigma \left(x\right)$ represent the predicted mean and standard deviation, respectively, and α is a constant which was set to α=1 for this instance. Additionally, the number of latent function samplings for interpretation was set at 500. The results are shown in Figure 14. The left half of the atom is colored according to the values of ${IG}_{i}^{\mu }\left(x\right)$ +${IG}_{i}^{\sigma }\left(x\right)$ , while the right half is colored based on ${IG}_{i}^{\mu }\left(x\right)$ ‐${IG}_{i}^{\sigma }\left(x\right)$ . If both halves of the atom are the same color, it suggests that there is confidence in the interpretability associated with that atom. In the ninth round of exploration, the impact of the phenylmethyl group was unclear, prompting further evaluation. The tenth round then focused on investigating the unclear effects of the phenol structure. Given that 2‐methylphenol has high solubility in water, it can be interpreted that the model inferred that phenol structures contribute to increased water solubility. Consequently, the eleventh and twelfth searches were interpreted as the model seeking compounds with phenol structures. By interpreting how the model conducts exploration during Bayesian optimization using this method, it becomes possible to determine the optimal timing to stop.

**Figure 15:**
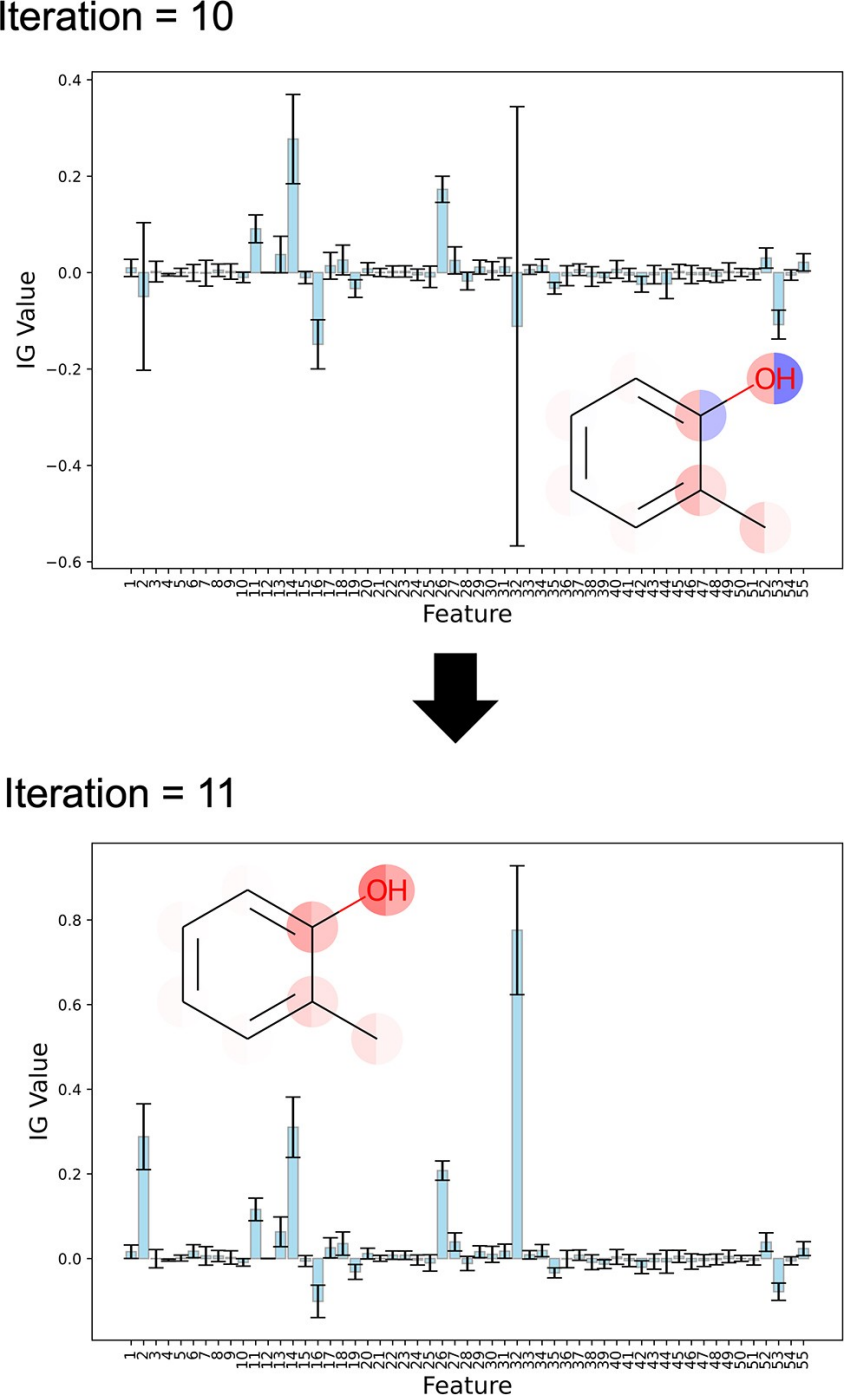
Interpret 2‐Methylphenol using models from iteration=10 and 11. The bar plot represents the ${IG}_{i}^{\mu }\left(x\right)$ of each feature, while the error bars indicate ${IG}_{i}^{\sigma }\left(x\right)$ .

Furthermore, the results of interpreting 2‐Methylphenol using the models from iteration=10 and 11 are shown in Figure 15. From these results, it can be observed that as the amount of training data increases, the interpretation results change significantly, leading to a reduction in prediction uncertainty. This confirms that the proposed method is effective not only in interpreting the predicted mean but also in accurately interpreting the predicted standard deviation.

## 5 Conclusions

In this study, we demonstrated that by sampling latent functions from a Gaussian Process model and applying the IG, we can interpret the predicted mean and predicted standard deviation. This methodology is not only applicable to GPR but also to DKLGPR, as well as to SVGPR and SVGPC, which can reduce computational costs, thereby offering a wide range of applicability. While this study provided an example of the property prediction of compounds, there are other fields where predictive reliability is crucial, such as medical image diagnosis. By interpreting models that combine Convolutional Neural Networks and GP models with this method, we can understand the risks associated with machine learning models more accurately. For instance, when identifying critical features during interpretation, employing this technique to calculate the contribution to predicted standard deviation enables us to establish the reliability of interpretation results: if the ${IG}_{i}^{\mu }\left(x\right)$ +${IG}_{i}^{\sigma }\left(x\right)$ and ${IG}_{i}^{\mu }\left(x\right)-{IG}_{i}^{\sigma }\left(x\right)$ for feature have the same sign, we can consider the interpretation trustworthy; otherwise, it is questionable. Additionally, if the predicted standard deviation is high, we should not rely on the prediction. Instead, we can identify features that significantly contribute to this standard deviation and consider collecting more data or retraining the model to reduce the predictive uncertainty. Furthermore, Bayesian optimization using GPR is a method that efficiently searches for optimal solutions by switching between exploration and exploitation, yet deciding when to stop can be challenging. By applying the proposed method to Bayesian optimization and interpreting it, we can better understand how the search is being conducted and make it easier to determine the optimal timing to stop, which proves to be useful.

## Conflict of Interests

The authors declare no conflicts of interest

6

## Data Availability

The data that support the findings of this study are available in reference number 10.1021/ci034243x. These data were derived from the following resources available in the public domain: https://github.com/deepchem/deepchem. The code is available at https://github.com/bbbccc88/Interpret‐Gaussian‐Process‐Models‐by‐using‐Integrated‐Gradients
